# Retrograde Gastric Intussusception

**DOI:** 10.4274/balkanmedj.2016.0828

**Published:** 2017-03-28

**Authors:** Ural Koç, Pınar Karakaş

**Affiliations:** 1 Department of Radiology, Erzincan University Mengücek Gazi Training and Research Hospital, Erzincan, Turkey; 2 Clinic of Pediatric Radiology, UCSF Benioff Children’s Hospital, Oakland, California

A 15-year-old boy presented to the emergency department with progressive emesis and epigastric pain for two days. Two days earlier, after fairly strenuous exertion, he developed acute epigastric pain radiating to his back and started vomiting. The emesis was initially food material but progressed into dark blood. He had a history of laparoscopic fundoplication and myotomy for achalasia ten years ago. Due to his persistent bolus sensation and difficulty swallowing, an upper gastrointestinal contrast study (Philips Medical Systems 64, Eindhoven, Holland) was performed and revealed a large filling defect causing obstruction at the distal oesophagus together with proximal oesophageal dilatation (Figure 1a. lateral, Figure 1b. anterior posterior view; purple arrow shows the filling defect). Contrast enhanced-computed tomography (CE-CT) showed all layers of the stomach and omental fat herniated into the lower oesophagus through a hiatal hernia (Figure 2a-d. axial view; Figure 3a-d. sagittal view; pink arrows show stomach layers, blue stars omental fat). CE-CT scanning was performed in the arterial phase during the first 25-30 seconds of the injection.

Intussusception is commonly seen in the small bowel and colon; however, such a process is not ordinarily seen in the gastroesophageal region in children (1). Retrograde gastric intussusception is a rare type of intussusception where a part of the stomach with all layers invaginates into the oesophagus (2). Hiatal hernia is commonly seen with retrograde gastric intussusceptions (2). Predisposing factors may involve increased gastric mobility due to ligamental and omental laxity, increased intra-abdominal pressure, high negative intra-thoracic pressure during inspiration due to physical exertion and prior history of myotomy and fundoplication (1,2,3,4). Retrograde gastric intussusception is an uncommon complication of prior myotomy operation for achalasia. Our paediatric patient had multiple risk factors including prior history of operation for achalasia and sudden increase in intra-abdominal pressure due to strenuous workout, which likely resulted in retrograde gastric intussusception. In this unusual case appropriate imaging and prompt clinical suspicion were crucial in making the correct diagnosis. Furthermore, preoperative diagnosis of retrograde gastric intussusception is very important in that it may lead to non-operative reduction or minimally invasive surgery instead of more invasive surgery (1). In our patient reduction of hiatal hernia, gastric intussusception and closure of diaphragmatic crura was laparoscopically performed.

## Figures and Tables

**Figure 1:**
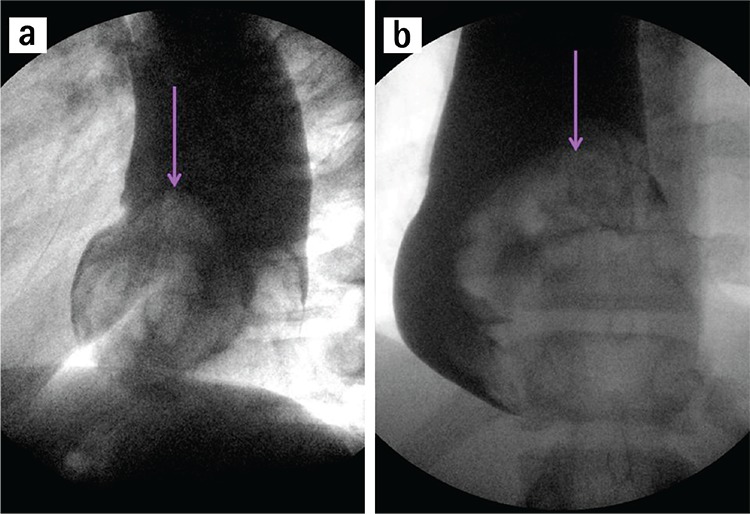
Lateral view (a), antero-posterior view; an upper gastrointestinal contrast study, a purple arrow shows a large filling defect causing obstruction at the distal oesophagus and proximal oesophageal dilatation (the filling defect) (b).

**Figure 2:**
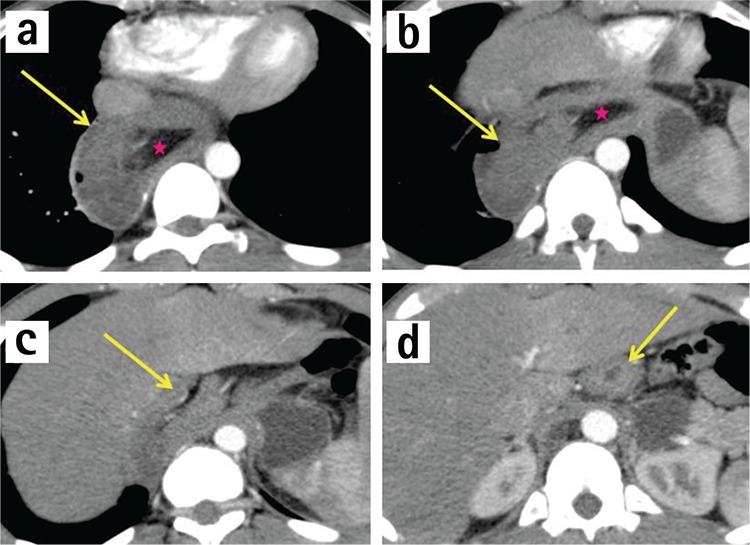
a-d. Axial view, (yellow arrows show stomach layers, red stars omental fat). Contrast-enhanced computed tomography (arterial phase) showed all layers of the stomach and omental fat herniated into the lower oesophagus through a hiatal hernia.

**Figure 3:**
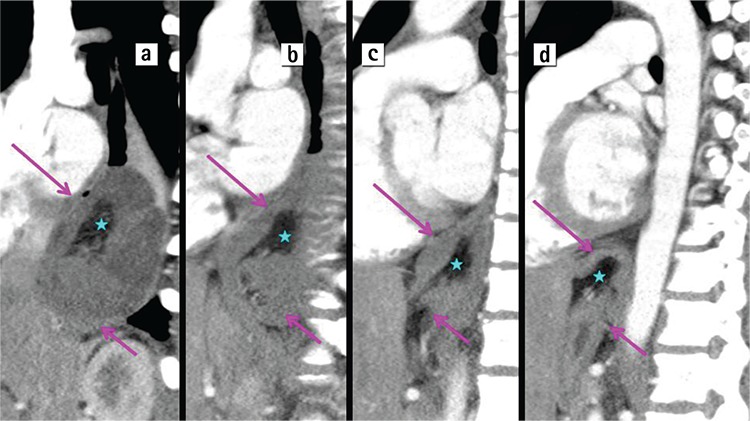
a-d. Sagittal view, (pink arrows show stomach layers, blue stars omental fat). Contrast-enhanced computed tomography showed all layers of the stomach and omental fat herniated into the lower oesophagus through a hiatal hernia.
